# *In Vivo* Monitoring of Angiogenesis Inhibition via Down-Regulation of Mir-21 in a VEGFR2-Luc Murine Breast Cancer Model Using Bioluminescent Imaging

**DOI:** 10.1371/journal.pone.0071472

**Published:** 2013-08-08

**Authors:** Dongliang Zhao, Yingfeng Tu, Lin Wan, Lihong Bu, Tao Huang, Xilin Sun, Kai Wang, Baozhong Shen

**Affiliations:** 1 Department of Radiology, the Fourth Hospital of Harbin Medical University, Harbin, Heilongjiang, China; 2 Key Laboratory of Molecular Imaging, College of Heilongjiang Province, Harbin, Heilongjiang, China; 3 Department of Cardiology, the Fourth Hospital of Harbin Medical University, Harbin, Heilongjiang, China; University of Frankfurt - University Hospital Frankfurt, Germany

## Abstract

MicroRNA-21 (miR-21) is overexpressed in a wide range of cancers and involved in tumor proliferation and metastasis. However, the potential function of miR-21 in regulating tumor angiogenesis has been little disclosed. In this study, we treated the cultured 4T1 murine breast cancer cells and human umbilical vein endothelial cells (HUVECs) with miR-21 mimic, antagomir-21 or negative control (scramble), which were subjected to MTT, terminal deoxynucleotidyl transferase dUTP nick end labeling (TUNEL), quantitative Reverse Transcriptase PCR (qRT-PCR) and immunoblotting analysis. In addition, 4T1 cells were implanted beneath the right breast fat pad of the VEGFR2-luc transgenic mice, which were randomly divided into three groups and received saline, antagomir-21 or scramble treatment once respectively after tumor model establishment. Bioluminescent imaging was used to monitor tumor growth and angiogenesis *in vivo* at 0d, 3d, 5d, 7d, 10d, and 14d after treatment. Mice were killed at the end of study and tumor tissues were collected for use. The results showed that knockdown of miR-21 by antagomir-21 decreased cell proliferation and induced apoptosis via targeting PTEN both in 4T1 cells and HUVECs. We also found the anti-angiogenesis and anti-tumor effects of antagomir-21 in the VEGFR2-luc transgenic mouse model using bioluminescent imaging. Moreover, the Western blotting data revealed that antagomir-21 inhibited tumor angiogenesis through suppressing HIF-1α/VEGF/VEGFR2-associated signaling pathway. In conclusion, the results from current study demonstrate that antagomir-21 can effectively suppress tumor growth and angiogenesis in VEGFR2-luc mouse breast tumor model and bioluminescent imaging can be used as a tool for noninvasively and continuously monitoring tumor angiogenesis *in vivo*.

## Introduction

Tumor angiogenesis is a vital process resulting in the formation of new blood vessels, which has been identified to play a critical role in tumor growth, invasion, and metastasis in recent cancer researches [1]. Generally, tumors cannot grow beyond 1∼2 mm^3^ in diameter without formation of new vessels to transport oxygen and nutrients [2]. During tumor angiogenesis, the activation of hypoxia-inducible factor-1α (HIF-1α) in hypoxia cells firstly triggers vascular endothelial growth factor (VEGF) expression, which stimulates vascular growth within hypoxic tumor tissues. Recent research has demonstrated that HIF-1α/VEGF/VEGFR2 signaling pathway is involved in endothelial cell proliferation, differentiation, migration, as well as vascular permeability [3]. A growing body of evidence has proved that inhibition of neovascularization via suppressing HIF-1α/VEGF/VEGFR2 signaling pathway may delay tumor progression and perhaps even starve tumor cells to death [4].

MicroRNAs (miRNAs), as a notable class of gene regulators, are a family of endogenous small single-strand RNAs with a length between 21 and 25 nucleotides. Recent studies showed that miRNAs act as tumor suppressors or oncogenes and play critical roles in tumor cells proliferation and apoptosis [5], [6]. Furthermore, growing evidence has shown that miRNAs can regulate the angiogenic process either through a direct effect on the endothelial cells or through an indirect effect on the tumor cells [7]. For example, miR-126 can promote blood vessel development by targeting negative regulators of MAP kinase and PI3K signaling pathways [8]. MiR-221/222 can inhibit angiogenesis by repressing c-kit in human umbilical vein endothelial cells (HUVECs) [9]. MiR-15b and miR-16 are involved in tumor angiogenesis via regulating VEGF expression [10]. And miR-17-92 cluster has also been identified as a negative regulator of angiogenesis *in vitro* and *in vivo* [11]. MiR-21 is one of the well characterized miRNAs, which is overexpressed in many kinds of tumors, especially in breast tumor [12]. Knockdown of miR-21 in MCF-7 cells could markedly suppress tumor proliferation and metastasis, indicating that miR-21 plays an important role in breast tumor development [13]. However, few investigations have been focused on the role of miR-21 and its target genes in breast tumor angiogenesis. Among the different genes reported to be targets of miR-21, PTEN has been identified as one angiogenesis modulator [14]. For example, Jiang *et al.* demonstrated that tumor angiogenesis can be triggered by PTEN or p53 tumor suppressor gene mutation [15]. In addition, Liu and his cooperators proved that down-regulation of miR-21 can decrease the expression of VEGF in prostate tumor [16]. Recently, miR-21 has been demonstrated to modulate apoptosis in HUVECs by down-regulation of PTEN [17]. Taken together, these data support that miR-21 is closely related to tumor angiogenesis, which prompts us to disclose the underlying role of miR-21 in breast angiogenesis and tumor growth.

Inhibition of angiogenesis has been proved to be an effective strategy to inhibit tumor growth. However, one of the challenges to use anti-angiogenic therapy is lacking of available strategy to noninvasively monitor angiogenesis of tumor *in vivo* and evaluate the treatment efficacy of chemotherapeutic drugs. Conventional methods to monitor angiogenesis are inconvenient, time-consuming and cannot be repeated, as it usually requires to sacrifice the animal model and to visualize microvessels by immunohistochemical staining of endothelial cell-specific markers. Therefore, there is an urgent need to develop a novel and noninvasive method to advance this field. Recently, bioluminescent imaging (BLI) allows us to detect vascular alteration in response to anti-angiogenic therapy conveniently and continuously for this imaging technique can provide a noninvasive and efficient observation of tumor growth and vasculature *in vivo*. In this current study, we used VEGFR2-luc transgenic mice as experimental animals, in which the VEGFR2 promoter drives the expression of luciferase. It is possible now to monitor the VEGFR2 expression in VEGFR2-luc mice using BLI, so we can visualize angiogenesis of tumor *in vivo* noninvasively and continuously [18].

The aims of the present study are to determine the role of miR-21 involved in breast tumor growth and angiogenesis and evaluate the effect of antagomir-21 in VEGFR2-luc mouse breast cancer model by BLI. We firstly performed experiments *in vitro* to demonstrate the anti-apoptotic role of miR-21 in 4T1 cells and HUVECs. From our data, knockdown of miR-21 expression with antagomir-21 treatment could obviously suppress proliferation and induce apoptosis via targeting PTEN in 4T1 cells and HUVECs. The data generated from experiments *in vivo* revealed angiogenesis inhibition mediated by antagomir-21 can be monitored using BLI. In addition, we further demonstrated that antagomir-21 inhibits angiogenesis by suppressing the HIF-1α/VEGF/VEGFR2 signaling pathway in breast tumor. This study indicated that manipulation of the expression of miR-21 represents a promising therapeutic strategy for breast cancer.

## Materials and Methods

### Animals

The study was approved by the ethical committee of Harbin Medical University, China. All animal studies were performed under the National Institutes of Health Guidelines for the care and use of experimental animals (NIH Publication No. 85–23, revised 1996). VEGFR2-luc mice were used as experiment animals, which initially purchased from Xenogen Company, bred in our animal facility to generate offspring. Transgenic VEGFR2-luc mice were created by using a gene-targeting approach that “knocked in” firefly luciferase cDNA into the first exon of the endogenous VEGFR2 locus in murine 129/SvEv embryonic stem cells. The expression of VEGFR2 can be able to drive the expression of luciferase gene.

### Cell Culture

Murine 4T1 breast cancer cells were purchased from ATCC and cultured in RPMI 1640 medium with fetal bovine serum (FBS) at a final concentration of 10%. The HUVECs were also purchased from ATCC and cultured in medium 199 (Gibco/Invitrogen), supplemented with 20% FBS, 50 µg/mL Endothelial Cell Growth Supplement (ECGS) (BD Sciences), 100 µg/mL Heparin (Sigma) and 1% penicilin–streptomycin. Cells were all cultured at 37°C in 5% CO_2_ incubator.

### Oligo Transfection, miR-21 Knockdown in Cultured Murine 4T1 Cells and HUVECs

The cells were seeded in antibiotic-free medium for 24 h prior to transfection. For up-regulation of miR-21, the cells were transfected with miR-21 mimic (GenePharma Co. Ltd.) using Lipofectamine 2000 (Invitrogen, USA) in serum-free Opti-MEM medium according to the manufacturer’s instructions. Transfection complexes were added to medium at final oligonucleotide concentration of 50 nM. For knockdown of miR-21, chemically modified antagomir-21 was used to inhibit miR-21 expression. The antagomir-21 sequence was 5′-UCAACAUCAGUCUGAUAAGCUA-3′. Chemically modified oligonucleotides 5′-AACAUCAGUCUGAUAAGCUAUU-3′ were used as a negative control (scramble). Antagomir-21 and scramble were also purchased from GenePharma Co. Ltd. 4T1 cells and HUVECs were transfected with antagomir-21 or scramble at a final concentration of 50 nM using Lipofectamine 2000 according to the manufacturer’s instructions. Opti-MEM medium was replaced 4 h post-transfection with the regular culture medium and incubated for another 48 h.

### Evaluation of Cell Proliferation using the MTT Assay

4T1 cells and HUVECs were plated in 96-well plates and transfected with miR-21 mimic, antagomir-21, and scramble respectively for 4 h. After the transfection, the serum-free medium was removed and cells were cultured with regular culture medium for another 48 h. To monitor cell survival, 4T1 cells and HUVECs in each well were incubated for 4 h with 0.5 mg/mL of MTT (Sigma). Thereafter MTT solution was removed and the formazan crystals trapped in cells dissolved in 150 µL of sterile DMSO (Sigma) by incubating at 37°C for 15 min. Absorbance was recorded at 490 nm using an Easy Reader 340 AT (SLT-Lab Instruments). Relative cell survival was calculated by setting control absorbance from untreated cells as 100%. Experiments were performed in triplicate.

### Orthotopic Xenograft Models and Treatment Protocol

Mice were raised under specific pathogen-free conditions in facilities and used in accordance with institutional guidelines when they grew up to 8–12 weeks old. To establish breast tumor model, 4T1 cells were firstly detached by a brief exposure to 0.25% trypsin and 0.02% EDTA. Trypsinization was terminated with medium containing 10% FBS. Then cells were collected by centrifugation and resuspended in phosphate-buffered saline (PBS) solution. Finally, five million cells (5×10^6^ cells, 100 µL) were subcutaneously injected beneath the right axillary mammary gland fat pad of the female VEGFR2-luc mice, which were anesthetized with 1–3% isoflurane gas. Tumors were allowed to grow up for 1 week and tumor volumes were measured with caliper. Tumor volume was calculated using the formula: V = (a×b^2^)/2, where “a” is the longest diameter, “b” is the shortest one.

A recent study showed that injection of antagomir markedly reduces corresponding miRNA levels in most normal murine tissues, and the silencing effect can last for over three weeks [19]. In order to evaluate the role of antagomir-21 in tumor angiogenesis inhibition, the tumor-bearing mice were randomly divided into three groups as follows: control group, antagomir-21 treatment group and scramble treatment group. To knockdown the expression of miR-21 in tumor in VEGFR2-luc transgenic mouse model, intratumor injections were processed with antagomir-21 or scramble through a 26-gauge needle (50 mg/kg antagomir-21 or scramble dissolved in 100 µL mixed solution) according to the manufacturer’s instructions. The mice of control group were received the same volume of saline. Intratumor injections were made in approximately 5 sites to make sure the drug diffusion evenly.

### Bioluminescent Imaging *in vivo*

BLI was performed using a highly sensitive, cooled charge-coupled device camera mounted in a light-tight specimen box (IVIS 200, Xenogen), with protocols similar to those described previously [20]. For imaging *in vivo*, mice were anesthetized with isoflurane and injected intraperitoneally with substrate D-luciferin (Invitrogen) at 150 mg/kg body weight. Optical signal intensity of the VEGFR2-luc mouse was acquired with 1 min of exposure time at 10 min after D-luciferin administration. Regions of interest (ROI) from displayed images were identified on the tumor sites and quantified as photons per second (p/s) using Living Image® software. Mice were killed at the end of the study and the tumor tissues were collected in 4% paraformaldehyde or liquid nitrogen for use.

### Quantitative Reverse Transcription-PCR (qRT-PCR)

Total RNA samples from the 4T1 cells, HUVECs and tumor tissues were isolated using Trizol reagent (Invitrogen) according to manufacturer’s protocols. Total RNA (1 µg) was reverse transcribed using High-Capacity cDNA Reverse Transcription Kit (Applied Biosystems) to obtain cDNA. The SYBR Green PCR Master Mix Kit (Applied Biosystems) was used in qRT-PCR to quantify the level of miR-21, with U6 as an internal control. The qRT-PCR was performed on 7500 FAST Real-Time PCR System (Applied Biosystems, USA) for 40 cycles. The primers were designed as follows: mmu-miR-21 sense primer: 5′-GGGGTAGCTTATCAGACTG-3′, mmu-miR-21 antisense primer: 5′-TGGAGTCGGCAATTGCACTG-3′. The primers for U6 are: sense primer: 5′-GCTTCGGCACATATACTAAAAT-3′, antisense primer: 5′-CGCTTCACGAATTTGCGTGTCAT-3′.

### Immunofluorescence Staining Analysis

Tumors were sectioned into 5 µm cryosections and prepared for use. For CD31 staining, sections were air dried firstly, then incubated in 4% paraformaldehyde to fix for 30 min at room temperature, washed 3 times by using PBS (every time for 5 min), following blocked by protein-blocking (5% normal horse serum in PBS) solution for 10 min. Drained protein block, sections were incubated with anti-CD31 antibody (1∶300 dilution, Abcam) for 2 h, washed with PBS, then incubated with Cy3-conjugated secondary antibody (1∶500 dilution, Invitrogen) for 1 h at 37°C. After staining with anti-CD31 antibody, sections were incubated with anti-CD61 antibody (1∶100 dilution, Abcam) for 2 h, and then incubated with Alexa Fluor 488-conjugated secondary antibody (1∶300 dilution, Jackson Immunoresearch) for 1 h at 37°C. Finally, cell nuclei were stained with 4′, 6-diamidino-2-phenylindole (DAPI) (Sigma) for 10 min.

A similar technique was applied for VEGFR2 staining. The slides were incubated over night at 4°C with the primary antibody (Cell Signaling) diluted at 1∶500, and incubated with Alexa Fluor 488-conjugated goat anti-rabbit secondary antibody (Jackson Immunoresearch) diluted at 1∶300 for 1 h at 37°C. Subsequently, cell nuclei were stained with DAPI for 10 min. Stained slides were stored on a flat surface at 4°C in the dark until imaged. Tissue images were captured with fluorescence microscope (IX71+DP72, Olympus).

### Blood Vessel Assessment

Microvessel density (MVD) of tumor tissue was evaluated by immunofluorescence double staining of CD31 and CD61. Ten random fields of each slide were analyzed at a ×200 magnification, excluding necrotic area. Microvessels were quantified according to a method described previously [21]. Images of each field were captured under filters representing the excitation wavelengths of red (expressing CD31 only) and green (expressing CD61 only), CD31 and CD61 merged images showed yellow. Average MVD was calculated by counting the average number of both CD31 and CD61 labeled vessels from ten randomized fields under the fluorescence microscope. All quantitative evaluations were carried out by ImagePro Plus software (version 6.0, Media Cybernetics).

### TUNEL Analysis

Apoptosis-induced DNA fragmentation was determined using the transferase mediated deoxyuridine triphosphate (dUTP)-digoxigenin nick end-labeling (TUNEL) assay. The 4T1 cells and HUVECs under different experimental conditions were fixed with 4% (w/v) paraformaldehyde and processed by using a commercial kit (Roche) in accordance with the manufacturer’s instructions. Tumor samples were fixed for 30 min at room temperature, rinsed with PBS, blocked for 10 min by 96% methanol mixed with 4% H_2_O_2_ at room temperature and permeabilized with 0.2% Triton X-100 in PBS for 5 min at 4°C. TUNEL staining was done using the in situ cell death detection kit (Roche) and the nuclei were stained with DAPI for 10 min. The numbers of TUNEL-positive cells and the total cells were captured with fluorescence microscope (IX71+DP72, Olympus) and cells apoptosis were determined with ImagePro Plus software.

### Western Blotting Analysis

Total protein samples were extracted from the cultured 4T1 cells, HUVECs and tumor tissues for protein immunoblotting, with the procedures essentially the same as described in details elsewhere [22]. Equal amounts of protein (100 µg) were fractionated by SDS-PAGE (8%–15% polyacrylamide gels) and blotted to PVDF membrane. Membranes were blocked for 2 h in 5% non-fat milk in Tris-buffered saline with Tween (TBST), then probed overnight at 4°C with the following primary antibodies respectively: PTEN (1∶200 dilution, Abcam), HIF-1α (1∶200 dilution, Cell Signaling), VEGF (1∶200 dilution, Abcam), VEGFR2 (1∶200 dilution, Cell Signaling), Caspase-3 (1∶500 dilution, Cell Signaling) and GAPDH (1∶5000 dilution, Cell Signaling). Membranes were then incubated with secondary antibody: Alexa Fluor® 800 goat anti-mouse or anti-rabbit IgG (Invitrogen) diluted at 1∶8000 at room temperature for 1 h. Western blot bands were captured by using the Odyssey Infrared Imaging System (LI-COR Biosciences) and quantified with Odyssey v1.2 software (LI-COR Biosciences), using GAPDH as an internal control. Western blotting experiments were repeated three times.

### Statistical Analyses

All quantitative data are expressed as the mean ± standard error of the mean (SEM). Statistical analyses were performed using the Student’s *t* test for comparisons of two groups and using one-way ANOVA for multi-group comparisons. Significance was set at *P*<0.05.

## Results

### Knockdown of miR-21 by Antagomir-21 Decreases Cell Proliferation and Induces Apoptosis in 4T1 Cells and HUVECs

**Figure 1:**
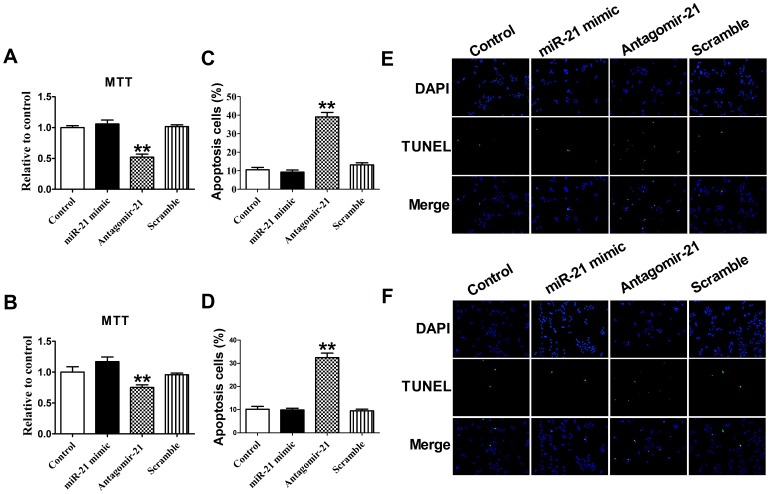
The effect of miR-21 antagomir on the cell proliferation and apoptosis. MTT assay of 4T1 cells (A) and HUVECs (B). Data are expressed as mean ± SEM, n = 3, **P<0.01 *vs* control. Quantification of apoptotic 4T1 cells (C) and HUVECs (D) were determined and shown in the diagram. The number of apoptotic cells was significantly increased in cells transfected with antagomir-21 than in control group. Data are expressed as mean ± SEM, n = 10, **P<0.01 *vs* control. (F). Apoptotic 4T1 cells (E) and HUVECs (F) were determined by TUNEL staining and visualized at 200× magnification. Green color is TUNEL staining representing apoptotic cell; blue color is the cell nucleus stained by DAPI.

We evaluated whether miR-21 contributes to 4T1 cells or HUVECs survival by blocking its expression with a specific antagomir. The 4T1 cells and HUVECs viability determined by MTT analysis were reduced significantly in cells upon transfection with miR-21 antagomir (*P*<0.05), but not with the miR-21 mimic and scramble, which did not affect the viability of 4T1 cells or HUVECs (*P>*0.05) (Figure 1 A and 1B).

**Figure 2:**
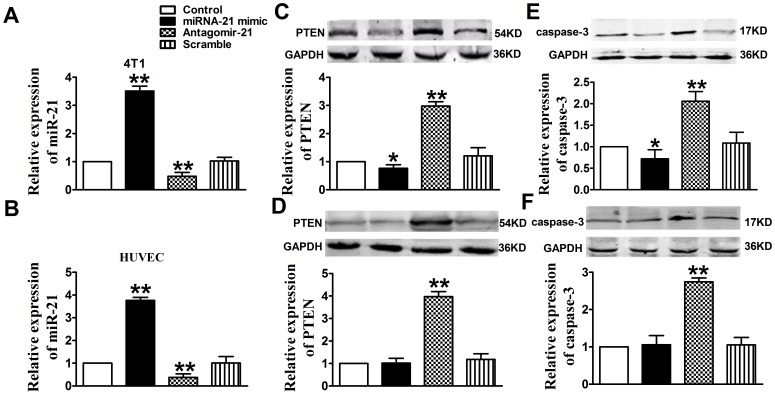
Down-regulation of miR-21 expression in 4T1 cells and HUVECs increased PTEN and Caspase-3 expression. Expression of miR-21 was showed in 4T1 cells (A) and HUVECs (B) that transfected with miR-21 mimic, antagomir-21, or scramble. Normal cells were used as control. Quantitative analysis of PTEN expression in 4T1 cells (C) and HUVECs (D). Quantitative analysis of Caspase-3 expression in 4T1 cells (E) and HUVECs (F). Proteins expression in normal cells, cells transfected with miR-21 mimic, antagomir-21 or scramble were examined by Western blotting. GADPH was used as an internal control. Data are mean ± SEM, n = 3, *P<0.05, **P<0.01, as compared with control cells.

In addition, the TUNEL staining results showed that transfection with miR-21 antagomir, but not with mimic, increased the percentage of TUNEL-positive cells both in the 4T1 cells and HUVECs, suggesting that miR-21 may play anti-apoptotic effect both in 4T1 cells and HUVECs (*P*<0.05) (Figure 1C–1F). Accordingly, we also found that, compared with control the expression of miR-21 was up-regulated with mimic transfection and down-regulated with antagomir-21 transfection in 4T1 cells and HUVECs (*P*<0.05), while the antagomir-21 scramble had no effect on miR-21 expression (*P>*0.05) (Figure 2A and 2B). Taken together, these data suggest that down-regulation of miR-21 may play pro-apoptotic effect both in 4T1 cells and HUVECs.

### Antagomir-21 Induces Apoptosis in 4T1 Cells and HUNECs via Targeting PTEN

MiR-21 has been shown to post-transcriptionally regulate the expression of the pro-apoptotic gene PTEN in human hepatocellular cancer [23]. Recently, miR-21 was demonstrated to attenuate apoptosis of HUVECs by down-regulation of PTEN [17]. In our study we also found that miR-21 is involved in regulating apoptosis in 4T1 cells and HUVECs via targeting PTEN. In Figure 2C and 2D, the data showed that knockdown of miR-21 with antagomir-21 induced overexpression of PTEN both in 4T1 cells and HUVECs (*P*<0.01), while scramble could not play the same role (*P>*0.05). In addition, Western blotting analysis demonstrated that Caspase-3 expression was increased in antagomir-21 treatment group (*P*<0.01), while not in scramble treatment group (*P>*0.05) (Figure 2E and 2F). Furthermore, the data showed that PTEN and Caspase-3 were decreased in miR-21 mimic treatment group only in 4T1 cells (*P*<0.05) (Figure 2C and 2E). Taken together, the above data demonstrated that antagomir-21 could induce apoptosis both in 4T1 cells and HUNECs via targeting PTEN.

### Antagomir-21 Inhibits Angiogenesis in VEGFR2-luc Mouse Breast Tumor Model Using BLI

**Figure 3:**
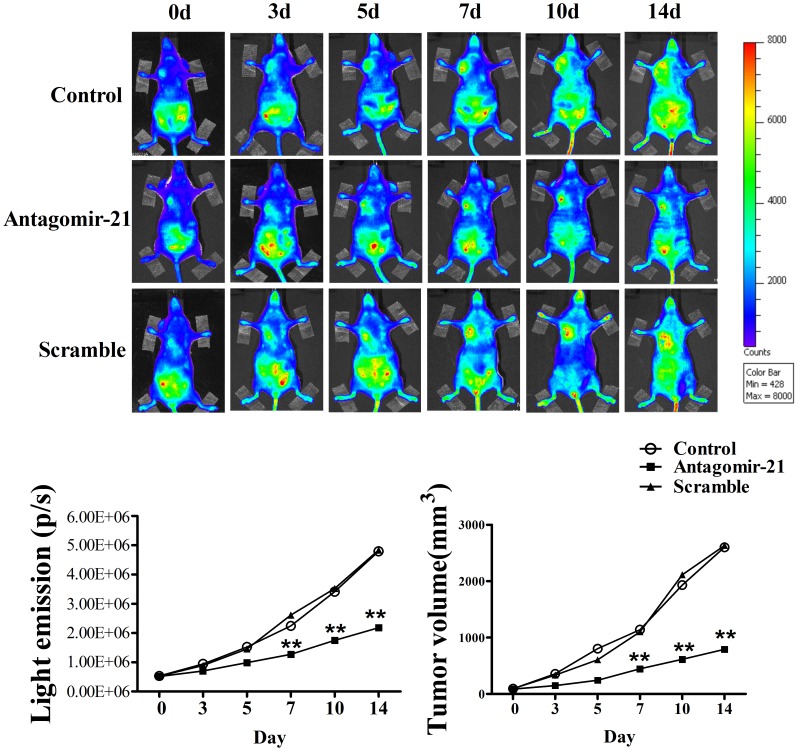
Bioluminescent imaging of tumor angiogenesis and change of tumor volumes. (A) Bioluminescent images of tumor-bearing mice were obtained at 0, 3, 5, 7, 10 and 14 d in the same imaging conditions. (B) The dynamic measurement of bioluminescent intensities in tumors treated with miR-21 mimic, antagomir-21 or scramble. Regions of interest (ROI) from displayed images were identified on the tumor sites and quantified as photons per second (p/s). (C) Tumor volume curves in mice treated with saline, antagomir-21 or scramble. Data are all shown as mean ± SEM. n = 5 in each group. **P<0.01, as compared with control.

In order to evaluate the effect of antagomir-21 in angiogenesis inhibition, we established VEGFR2-luc mouse breast tumor model, in which the expression of luciferase can be driven by VEGFR2 promoter and thus allows us to directly visualize angiogenesis using BLI *in vivo*. The BLI of this female VEGFR2-luc mouse is characterized by relatively low signal intensity in the majority of tissues except for the salivary glands and intra-abdominal reproductive organs like ovaries and uterus (Figure 3A). The data showed that there was no difference in average bioluminescent intensities among three groups at 0 day (Figure 3A and 3B). However, with the tumor development, tumor areas showed higher bioluminescent intensities in control (1.52×10^6^ p/s) and scramble (1.45×10^6^ p/s) treatment groups than antagomir-21 (0.96×10^6^ p/s) group 5 days after treatment (Figure 3A and 3B). Excitingly, at 14 days of treatment, the bioluminescent intensities in angatomir-21 treatment group unchanged significantly relative to 7th day of treatment, while the bioluminescent intensities increased gradually in control and scramble treatment groups (Figure 3A and 3B). These data suggest that antagomir-21 could suppress angiogenesis *in vivo*. In addition, VEGFR2-luc mice can be used as a noninvasive tool of performing real-time analysis to monitor the effect of antagomirs or pharmacological treatment in angiogenesis inhibition *in vivo*.

### Antagomir-21 Plays a Key Role in Inhibiting the Growth of Breast Cancer

To assess the function of antagomir-21 in inhibiting the growth of breast cancer, we continually measured the gross tumor volumes by caliper for 2 weeks. Before receiving treatment, tumor volumes were similar (nearly 100 mm^3^) among three groups. In Figure 3C, the results showed that tumor volumes of control group (1141.00±27.2 mm^3^) and scramble treatment group (1100.40±15.49 mm^3^) were significantly larger than antagomir-21 treatment group (446.42±4.42 mm^3^) at 7th day of treatment, while there was no difference in tumor volumes between saline and scramble groups (Figure 3C). With the extension of observation, we found that the tumor volumes in both saline and scramble treatment groups increased markedly, nearly to 2500 mm^3^ two weeks after treatment (Figure 3C). Though the tumor volumes of mice treated with antagomir-21 increased gradually during two weeks observation period, the growth trend (from 100 mm^3^ to 800 mm^3^) was not so fast as compared with saline and scramble groups (Figure 3C). The above data indicates antagomir-21 strongly retards breast tumor growth *in vivo*.

### Knockdown of miR-21 with Antagomir-21 Inhibits Tumor Angiogenesis through Immunofluorescence Analysis

**Figure 4:**
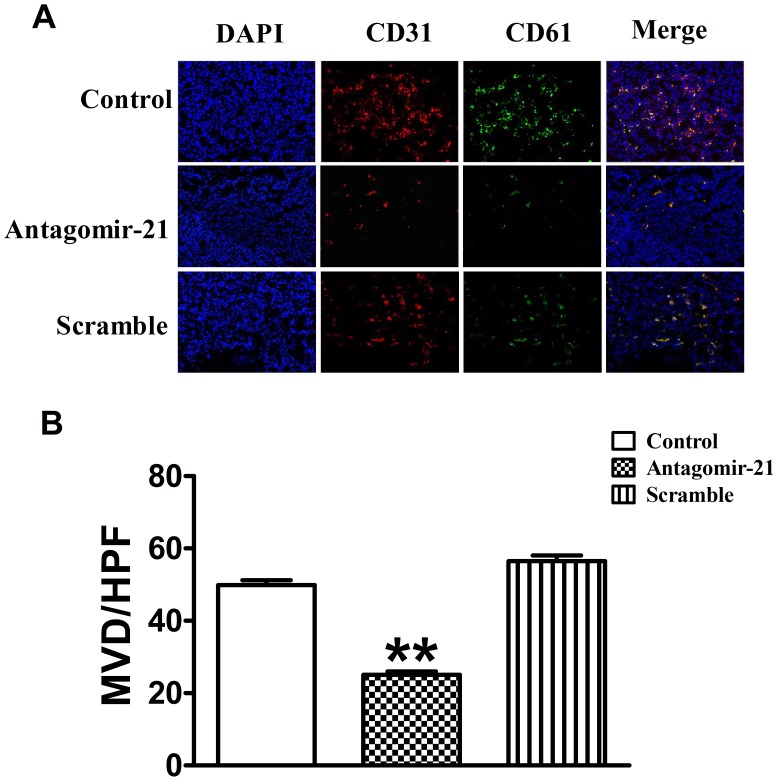
Knockdown of miR-21 expression inhibits angiogenesis of breast tumors. (A) Representative CD31 and CD61 immunostaining images were taken under different experimental conditions as indicated (Original magnification, ×200). Red color is staining for CD31, green color is staining for CD61, and blue color is staining for cell nucleus. (B) MVD of breast tumor was determined by the vessel counts, which were stained with antibodies against both CD31 and CD61. MVD: microvessel density, HPF: high-power field. Data are expressed as mean ± SEM. **P<0.01 *vs* control group.

To further confirm the role of antagomir-21 in angiogenesis inhibition in breast tumor, we stained the tumor slides with both anti-CD31 and anti-CD61 antibodies. The results of double immunofluorescence staining indicated that the number and diameter of vascular networks in sections treated with antagomir-21 were fewer and smaller compared to those treated with saline or scramble. MVD of breast tumor was determined by the vessel counts, which were stained with antibodies against both CD31 and CD61. In tumors treated with saline and scramble, MVD was (50±1.21) and (58.11±1.37) respectively, while MVD of those treated with antagomir-21 was only (25.22±0.74) (Figure 4).

**Figure 5:**
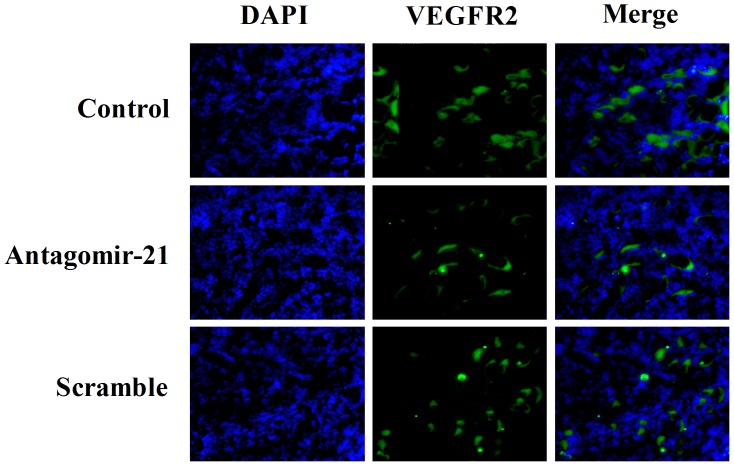
VEGFR2 immunostaining assay of tumor sections from different groups. Representative VEGFR2 immunostaining images were taken under conditions as indicated (original magnification, ×200). Immunostaining for VEGFR2 was green, cell nucleus was blue. The amount of stationary VEGFR2 staining was significantly lower in the antagomir-21 group relative to control group, while there was no difference between control group and scramble group.

Otherwise, we also evaluated the expression of VEGFR2 in mouse breast tumor, which was closely associated with tumor angiogenesis. Immunofluorescence staining with anti-VEGFR2 antibody showed low expression of VEGFR2 in tumors treated with antagomir-21 versus treated with saline or scramble (Figure 5), which was coincident with the results of BLI. Taken together, these results obtained from immunofluorescence staining indicated that antagomir-21 could significantly inhibit breast tumor angiogenesis *in vivo*.

### Down-regulation of miR-21 with Antagomir-21 induces Tumor Cell Apoptosis

**Figure 6:**
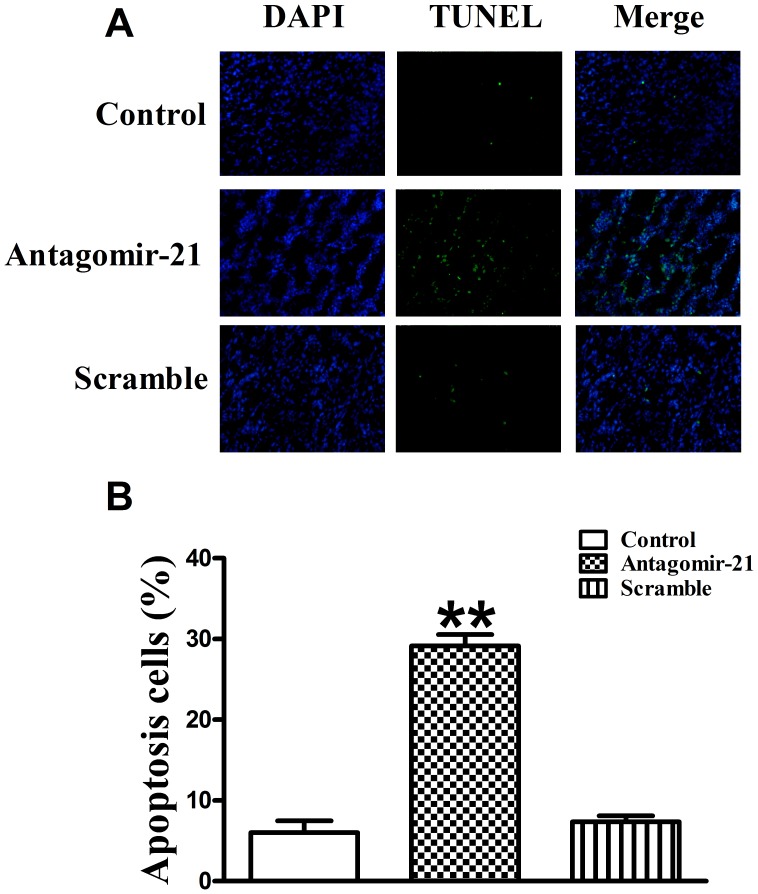
The effect of antagomir-21 on tumor cell apoptosis. Cell apoptosis was determined by TUNEL staining. (A) Green color is apoptotic cell stained by TUNEL; blue color is the cell nucleus stained by DAPI. Images were taken at a magnification of ×200. (B) Quantification of cell apoptosis from conditions in A. Data are expressed as mean ± SEM, n = 10, **P<0.01 *vs* control group.

To evaluate the extent of apoptosis in tumor tissues, apoptotic cells were stained using the TUNEL method. The number of apoptotic-positive cells was counted in a high-power field (×200 magnification). A notable increase of apoptotic-positive cells was observed in the antagomir-21 treated tumor, not scramble treated, compared with the saline control (Figure 6A). Data of apoptotic indexes of the three groups were shown as follows: control group (6.03±1.44%), antagomir-21 group (29.15±1.40%) and scramble group (7.34±0.77%) (Figure 6B). These data suggest that suppression of miR-21 expression in breast tumor likely increases tumor cell apoptosis, supporting the notion that antagomir-21 has a therapeutic potential.

### Antagomir-21 Inhibits Tumor Angiogenesis through Targeting HIF-1α/VEGF/VEGFR2 Signaling Pathway

**Figure 7:**
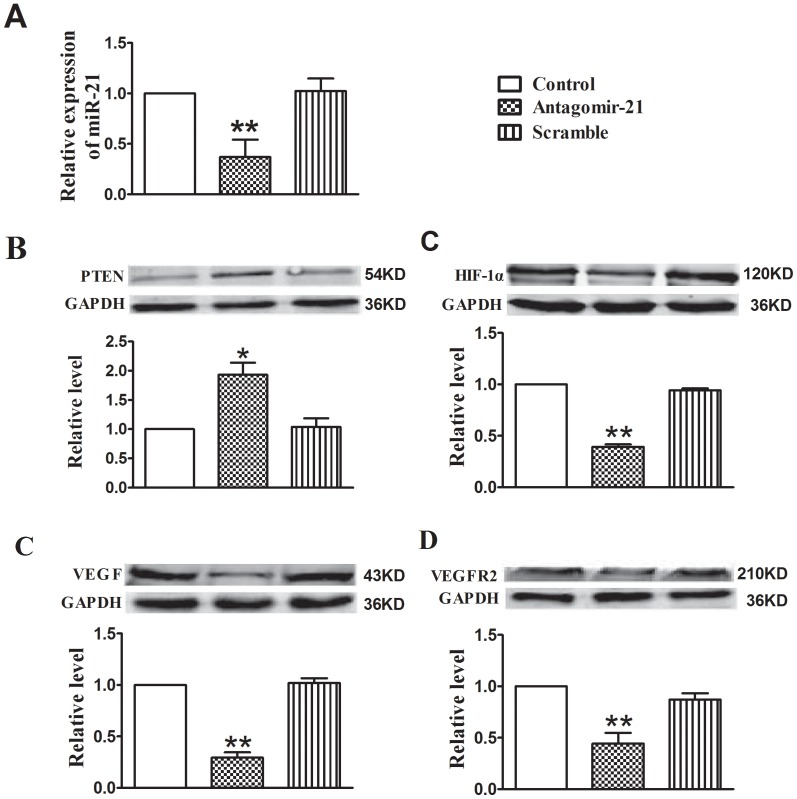
Down-regulation of miR-21 with antagomir-21 inhibits angiogenesis through targeting HIF-1α/VEGF/VEGFR2 signaling pathway. (A)Expression levels of miR-21 in tumors treated with miR-21 mimic, antagomir-21 or scramble. Data are expressed as mean ± SEM. **P<0.01 *vs* control group. (B–E) Protein levels of PTEN, HIF-1α, VEGF and VEGFR2 were analyzed by Western blotting assay. Data are expressed as mean ± SEM. n = 3 in each group. *P<0.05 *vs* control group, **P<0.01 *vs* control group.

Two weeks after antagomir-21 treatment, using qRT-PCR assay, we found that miR-21 expression was markedly inhibited by antagomir-21 relative to control. However, the expression of miR-21 could not be suppressed by scramble treatment (Figure 7A). Concomitantly, the production of PTEN was enhanced by miR-21 down-regulation, contrarily the expression of HIF-1α, VEGF and VEGFR2 were markedly decreased by antagomir-21, while the scramble could not produce the same effects in angiogenesis inhibition (Figure 7B–7E). The above results reveal that down-regulation of miR-21 with antagmir-21 inhibits angiogenesis in breast tumor via targeting HIF-1α/VEGF/VEGFR2-associated signaling pathway.

## Discussion

The major findings of our study are as follows: firstly, we demonstrated that antagomir-21 could down-regulate the miR-21 expression in 4T1 cells and HUVECs; secondly, we found that antagomir-21 could suppress 4T1 cells proliferation *in vitro* and *in vivo*; thirdly, antagomir-21 induces 4T1 cells and HUVECs apoptosis via targeting PTEN. In addition, we found the feasibility to noninvasively monitor angiogenesis in VEGFR2-luc mouse breast tumor model. As a noninvasive molecular imaging strategy, BLI can effectively track and quantify the anti-angiogenesis treatment response of antagomir-21 in mouse tumor model. Moreover, we confirmed that antagomir-21 could suppress miR-21 expression in breast tumor tissues, which is involved in inhibiting angiogenesis by targeting HIF-1α/VEGF/VEGFR2 signaling pathway in breast cancer. Finally, we also found that antagomir-21 could induce tumor cell apoptosis in breast tumor *in vivo*, which was verified by TUNEL analysis. These findings not only help us understand the anti-angiogenesis and anti-tumor effects of antagomir-21 but also improve our understanding of miRNAs that may serve as potential therapeutic targets in tumor.

Noninvasive and continuous monitoring of tumor angiogenesis *in vivo* is a vital step to assess anti-angiogenic therapy strategy. To our knowledge, our research for the first time confirmed that BLI could be used as a direct and accurate method for observing dynamic change of VEGFR2 in mouse breast tumor model. Molecular imaging strategies, including contrast-enhanced ultrasound [24], positron emission tomography or single photon emission computed tomography [25], fluorescent proteins and bioluminescent [26], have all been used to image VEGFR2 *in vivo*. However, BLI is more sensitive, non-radioactive, and lack of the autofluorescence background compared with other imaging techniques. Transgenic and knock-in animals expressing luciferase under the VEGFR2 promoter have been successfully used to visualize wound healing and peritumoral angiogenesis [18], [27]. Accordingly, we successfully monitored the anti-angiogenesis effect of antagomir-21 in breast tumor development using VEGFR2-luc tumor-bearing mice. In the current study, we surprisingly observed that the bioluminescent intensities increased with development of the implanted tumors. However, bioluminescent intensities of tumors did not always correlate precisely with tumor volumes, indicating that angiogenesis during tumor development may be a dynamic rather than constitutive process. Furthermore, the overexpression of VEGFR2 in tumor growth could be abolished by antagomir-21 but not scramble treatment using BLI analysis. Taken together, we demonstrated that BLI may lay the foundation for development of noninvasive probes for monitoring similar molecular events in human tumors in future.

Breast cancer is characterized by rapid cellular proliferation, which leads to hypoxic conditions within the tumor and then hypoxic conditions trigger angiogenesis by releasing pre-angiogenesis factors, such as VEGF, to recruit new blood supplies. Therapeutic strategy via targeting VEGF/VEGFR signaling pathway has been used for the treatment of solid tumors, and many pre-clinical studies have reported successful application of anti-vascular agents particularly in combination with other therapies [28]. A growing body of evidence shows that miRNAs are involved in regulating angiogenesis via targeting HIF-1α/VEGF/VEGFR signaling pathway in tumor development *in vivo*. For example, Zhang et al. demonstrated that inhibition of miR-20b expression increases the protein levels of HIF-1α and VEGF in normoxic tumor cells. In contrast, overexpression of miR-20b in hypoxic tumor cells can decrease the protein levels of HIF-1α and VEGF [29]. Another famous study disclosed that growth factor-induced miR-296 contributes significantly to angiogenesis by directly targeting the hepatocyte growth factor-regulated tyrosine kinase substrate (HGS) mRNA leading to decrease levels of HGS and reduce HGS-mediated degradation of the VEGFR2 [30]. Recently, more attention is focused on the function of miR-21 in angiogenesis during tumor development. Liu and his cooperators demonstrated that inhibition of miR-21 can decrease HIF-1α and VEGF proteins expression in prostate cancer cells [16]. Coincidently, in the current study we also found that antagomir-21 played anti-angiogenesis effect via targeting HIF-1α/VEGF/VEGFR signaling pathway. Treated with antagomir-21, not scramble, the miR-21 expression was reduced significantly in tumor-bearing mouse model relative to control group (Figure 7A). Concomitantly, the production of HIF-1α, VEGF and VEGFR was decreased with antagomir-21 intervention. To further confirm antagomir-21 effect on angiogenesis, we also performed immunofluorescence staining for CD31, CD61 and VEGFR2 on tumor tissues. The results showed that the expression of CD31, CD61 and VEGFR2 was lower in antagomir-21 treatment group than saline or scramble treatment group, which was in accordance with the BLI analysis.

Anti-angiogenesis therapy is aimed at cutting the blood supply of a tumor, which may induce tumor cells apoptosis or destroy the tumor itself. MiR-21 has been demonstrated to play a role in regulating cell apoptosis through modulating target genes as Bcl-2 and PDCD4 [12]. In addition, recent evidence from breast cancer studies indicated that inhibition of miR-21 level leads to a significant decrease in tumor growth and an increase in Caspase activity [31]. In our study, we also found that knockdown of miR-21 with angatomir-21 could induce apoptosis both in 4T1 cells and HUNECs. Furthermore, we also elucidated that application of antagomir-21 not only played anti-angiogenesis role but also promoted tumor cells apoptosis in breast tumor therapy. Thus, blocking the action of miR-21 in breast tumor development implies tremendous therapeutic value.

Though this devised noninvasive imaging method allows us to examine VEGFR2 expression level and localization of VEGFR2 in vivo, it is hard to quantify the precise amount of VEGFR2 using this VEGFR2-luc transgenic mouse model. We found an interesting phenomenon that after treatment of two weeks, some mice showed tumor necrosis and bioluminescent intensity of these mice gradually decreased because of necrosis area lacking of image signal. Despite these potential limitations, the data from the current study shed new light on noninvasive imaging of angiogenesis inhibition in cancer research.

In conclusion, in this study we identified the anti-angiogenesis role of antagomir-21 in VEGFR2-luc mouse breast tumor model via noninvasive BLI and disclosed potential mechanism of antagomir-21 in inhibiting angiogenesis via suppressing HIF-1α/VEGF/VEGFR2 signaling pathway. These findings implicate that miRNAs may be able to serve as new targets for anti-tumor and anti-angiogenic therapy, which provide a new, individualized treatment strategy for cancer therapy. In addition, BLI strategy provides a unique and powerful methodology, and will be extremely valuable in the diagnosis and treatment application of clinical patients in the future.
